# Incomplete histological margins following planned narrow excision of canine appendicular soft tissue sarcomas and mast cell tumors, using the residual tumor classification scheme

**DOI:** 10.1111/vsu.13852

**Published:** 2022-07-13

**Authors:** David L. Haine, Rachel Pittaway, Davide Berlato, Jackie Demetriou

**Affiliations:** ^1^ Cave Veterinary Specialists, part of Linnaeus Veterinary Limited; ^2^ Dick White Referrals Cambridge UK; ^3^ AniCura Animal Oncology and Imaging Center Hünenberg Switzerland

## Abstract

**Objective:**

To describe the frequency of incomplete histological margins following planned narrow excision (PNE) of mast cell tumors (MCTs) and soft tissue sarcomas (STSs), and to assess the residual tumor classification (R) scheme for reporting histological margins in clinical cases.

**Study design:**

Retrospective clinical study.

**Sample population:**

Forty‐four client‐owned dogs with 47 masses.

**Methods:**

Medical records of dogs undergoing planned narrow excision of STSs and MCTs were reviewed (2016‐2019). Histologic specimens were reviewed by a single pathologist and assigned R scoring (histologically incomplete/R1 margins defined as “tumor on ink”).

**Results:**

Six out of 23 (26%) MCT PNEs and 10/42 (42%) of STS PNEs resulted in R1 margins. R1 margins were more likely when performing PNE with 6‐10 mm lateral measured surgical margins (LMSMs) versus 0‐5 mm LMSM for MCTs (1/14 vs 5/9), but not STSs (3/7 vs 7/17) (*P* = .049).

The R scheme resulted in higher retrospective percentage agreement in histological reporting than defining incomplete histological margin as tumor cells within ≤1 mm of the margin (83% vs 68% agreement). Complications occurred in 12/47 surgeries, with none requiring additional surgery. Tumors recurred in 3/18 (17%) STSs and 2/18 (11%) MCTs.

**Conclusion:**

Fewer R1 margins were obtained when PNE with LMSM of 6‐10 mm was performed for mast cell tumors. The use of the R scheme increased agreement in histopathological margin assessment.

**Clinical significance:**

Planned narrow excision is a viable technique for histopathological diagnosis of appendicular soft tissue sarcomas and mast cell tumors for limb salvage.

## 1 INTRODUCTION

Soft tissue sarcomas (STSs) and mast cell tumors (MCTs) are commonly encountered in small animal practice.^1^, ^2^, ^3^ The presentation and treatment decision‐making algorithm for the management of these tumors is often similar, although they are behaviorally and histologically distinct.^3^, ^4^ There is no clear consensus on approach; however, wide or proportional surgical margins (2 cm or proportional for grade I/II MCTs,^5^, ^6^, ^7^ 3 cm for high‐grade MCTs,^1^ 3 cm for STSs^2,8^) and a deep fascial plane are frequently cited as necessary for complete surgical excision. Unfortunately, when the presentation is on the limbs, obtaining these surgical margins is often impossible without considering either radical resection (eg, partial or complete limb amputation), or techniques potentially associated with additional costs and morbidity.^3^, ^9^, ^10^

One strategy to manage these tumors is marginal excision (ME): removing macroscopic disease, followed by active surveillance and/or adjunctive therapy based on histopathology.^11^, ^12^ This strategy has been shown to have good long‐term clinical outcomes in veterinary patients^11^, ^13^, ^14^ and is considered to be the standard of care for the treatment of soft‐tissue sarcomas in human medicine.^15^, ^16^ Marginal excision can be defined as the removal of a tumor on or just outside the pseudocapsule/grossly visible tumor (GVT); however, it is also frequently used in the literature as a synonym for excisional biopsy or incomplete excision,^11^, ^17^ where the principles of excision outside the GVT with a deep fascial plane have not have been adhered to, with a high likelihood of leaving residual disease.^11^, ^18^, ^19^, ^20^ In our experience, there is a subset of appendicular tumors where limb salvage is preferred and where a compromise is made to remove as much of the tumor and surrounding tissues as possible while maintaining a tension‐free closure. This approach may confer better oncologic results than a true marginal or intralesional excision. Standard practice at the authors' institution is to perform excision of appendicular STSs/MCTs while strictly adhering to oncological principles of excision outside the GVT and removing a deep fascial plane, followed by active surveillance. We define this as a planned narrow excision (PNE): removing as much tissue as possible within the constraints of the anatomical location and the need for primary wound closure (as healing by second intention would preclude/delay some adjuvant therapies). The authors' impression is that there is a clinical difference in histological margin attainment between PNE and ME, with PNE resulting in fewer recommendations for adjunctive therapy.

Incomplete histological excision of MCTs and STSs (the presence of tumor cells close to the histological margin) has been consistently associated with the prognosis for low‐grade tumors.^9^, ^17^, ^19^, ^21^, ^22^ However, the definition of what entails an “incomplete histological excision” varies between different studies (eg, tumor cells ≤1 mm,^5^, ^7^, ^17^ 2 mm,^23^ or 3 mm,^12^ from the inked surgical margin), meaning direct comparisons cannot be performed. The residual tumor classification (“R”) scheme is an objective measure of histopathological margin reporting, with a definition of “tumor on ink” to define an incomplete histological margin (R1), and all other margin lengths being designated as complete (R0). The use of this scheme has been recommended for use in veterinary oncology due to its simplicity and widespread use in human oncology, allowing better comparison of results between studies, and reducing the impact of processing artifacts on interpretation.^24^

The primary objectives of this study were to describe the frequency of incomplete histological margins following PNE of MCTs and STSs and to assess the R scheme for reporting histological margins in clinical cases. Secondary objectives were to assess whether patient signalment and the size of lateral surgical margins affect the percentage of incomplete histological margins within this population. Clinical outcomes following these surgeries will also be reported, including surgical complications, indications for adjunctive therapy, and recurrence of local or metastatic disease. The authors hypothesize that PNEs of STSs and MCTs will result in fewer R1 histological margins than reported in the literature for ME of these tumors and that the frequency of R1 margins would be associated with the size of lateral measured surgical margins. We also hypothesize that the use of the R scheme will improve interobserver agreement in margin reporting.

## 2 MATERIALS AND METHODS

Medical records at a single referral institution were reviewed to identify dogs that underwent PNE of appendicular STSs or MCTs between 2016 and 2019. Cases that received preoperative adjuvant treatment (eg, preoperative chemotherapy, or preoperative radiation therapy), and those in which the intent of the surgery was defined as “debulking” or “intralesional,” or the intent was to excise >10 mm outside the GVT, were excluded. “Appendicular” was defined as localized at, or distal to, the stifle or elbow joints. A PNE was defined as the surgeon′s intention to resect outside the GVT with ≤10 mm lateral surgical margins and a deep fascial plane. Data extracted from patient records included breed, weight, age, use of and type of preoperative imaging, description of surgery including adverse events (classified Grade 1‐5 in order of severity as suggested by Follette et al.),^25^ histopathological tumor characteristics (size, location, grade, and R scheme scoring), duration of hospitalization, and short‐term hospital follow up. For analysis of surgical margins, lateral measured surgical margins (LMSMs) outside the GVT were assigned to 1 of 2 groups, (0‐5, or 6‐10 mm) based on surgical reports. Radiotherapy reports attached to the patient′s clinical history (received following radiotherapy at 1 of 2 outside institutions) were also reviewed.

Veterinarians referring cases were contacted to obtain follow‐up information; questions were asked to ascertain the patient′s clinical status and progression of the disease. If the patient had died, then the date and cause of death (if known) were recorded. Follow‐up data were only considered for use if a minimum follow‐up period of 12 months was obtained; otherwise, they were considered absent. Data from cases where no follow‐up was available were used for the primary study objective; however, these cases did not contribute toward clinical outcome reporting. Dogs that were designated as lost to follow‐up, which died of causes other than the tumor, or that were still alive without evidence of recurrence or metastasis were censored at the point of completion of data acquisition (September 2020). The proportion of cases considered “clinically indicated” for adjunctive therapy was calculated by summating the number of surgeries that returned R1 margins, and/or high‐grade tumors, regardless of whether adjunctive therapy was subsequently performed.

Hematoxylin‐and‐eosin (HE) stained sections from all cases were reviewed by a single board‐certified pathologist, blinded to patient data, to confirm the histopathological diagnosis, provide a grade, and for margin evaluation. Margin evaluation was performed by assessment of radial sections taken at 5 points around the mass – 4 lateral and the deep margin. All tissue margins had been inked at the time of initial submission and before routine processing. Histological margins were reported as either R1 (tumor cells visible on the inked surgical margins), or R0 (histologically tumor free). The percentage of margins classified as incomplete, when an incomplete margin was defined as tumor cells ≤1 mm from the inked margin,^5^, ^7^, ^17^ was also calculated (referred to as ≤1 mm scheme). For comparison of interobserver agreement in histological margin reporting, the original contemporaneous histopathological reports were reviewed and assigned retrospectively both R scoring and ≤1 mm scheme scoring by an author (DH) based on the reported quantified surgical margin. Mast cell tumors were assigned a histologic grade both according to the 3‐tier Patnaik grading system^26^ and the 2‐tier Kiupel grading system.^27^ Soft tissue sarcomas were graded as low/intermediate/high (grade I‐III).^28^

With the hypothesis that there would be 50% R1 margins in the PNE group, taking a value of 76%^17^ as the frequency of incomplete histological margins following ME of STSs, with a significance level of 0.05 and a power of 0.8, it was calculated that 23 cases would be required in each of the STS and MCT groups to demonstrate a significant difference between PNE and historical marginal excision in the frequency of incomplete histological margins. The frequency of incomplete histologcal margins is reportedly higher following ME of MCTs (88%), therefore the lower value was used.^29^

Binary logistic regression with multiple explanatory variables was used to assess whether completeness of margins was influenced by (i) sex (4 categories), age, body weight, body condition score (treated as continuous), and tumor size; or (ii) tumor type (STS or MCT), surgical margin (0‐5 mm/6‐10 mm), and the interaction of tumor type and margin. Significance was assessed with likelihood ratio tests, and *P* values <.05 were considered significant.

The study methodology was approved by the RCVS ethics review panel.

## 3 RESULTS

A medical record search identified 95 appendicular STSs/MCTs surgically excised within the study period. Of these, 42 were excluded due to either intentional wide, or intentional intralesional excision. Two additional masses were excluded due to preoperative administration of chemotherapeutic agents, and an additional 4 were excluded due to unclear diagnoses or slides not being available for review. This left 44 eligible patients with 47 masses, comprising 23 MCTs and 24 STS. Surgical excisions were performed by 1 of 5 ACVS/ECVS boarded surgeons or a resident under the direct supervision of a boarded surgeon.

Eighteen breeds were represented, including mixed breed (*n* = 9), pug (*n* = 6), Labrador retriever (*n* = 5), English springer spaniel (*n* = 3), Staffordshire bull terrier (*n* = 3), whippet (*n* = 2), golden retriever (*n* = 2), boxer (*n* = 2), greyhound (*n* = 2), English cocker spaniel (*n* = 2), and 1 each of Weimaraner, French bulldog, Jack Russell terrier, Boston terrier, standard poodle, Siberian husky, Great Dane, and Airedale terrier. The median age was 7 years 9 months old (range 4 months to 14 years 0 months); the mean weight was 24.0 kg (± 13.4), median body condition score was 6 out of 9 (range 4‐9 out of 9). There were 21 female‐neutered, 16 male‐neutered, 5 male‐entire, and 2 female‐entire dogs.

All tumors underwent sampling before surgery for confirmation of tumor type. Of these, 6 were diagnosed by preoperative incisional biopsy (0/23 MCTs, 6/24 STSs), and the remaining 41 were diagnosed by needle aspiration and cytology (23/23 MCTs, 18/24 STSs). Tumor locations included antebrachium (9), pes (9), stifle (8), elbow (4), crus (3) carpus (3), and tarsus (1). Mean and median tumor diameters were 25 mm (± 16 mm) and 20 mm (range 3‐60) respectively. Details of preoperative staging are listed in Table 1. At the authors′ institution, splenic/hepatic aspirates were not routinely performed in the study period without evidence of pathology and were therefore not performed on any patients within the study. All staging was negative.

**TABLE 1:** Preoperative staging performed on patients within the study

	Lymph node cytology	Abdominal ultrasound	Thoracic radiographs	Computed Tomography	None
Mast cell tumors	20	18	0	1	4
Soft tissue sarcomas	5	5	10	6	5

**FIGURE 1:**
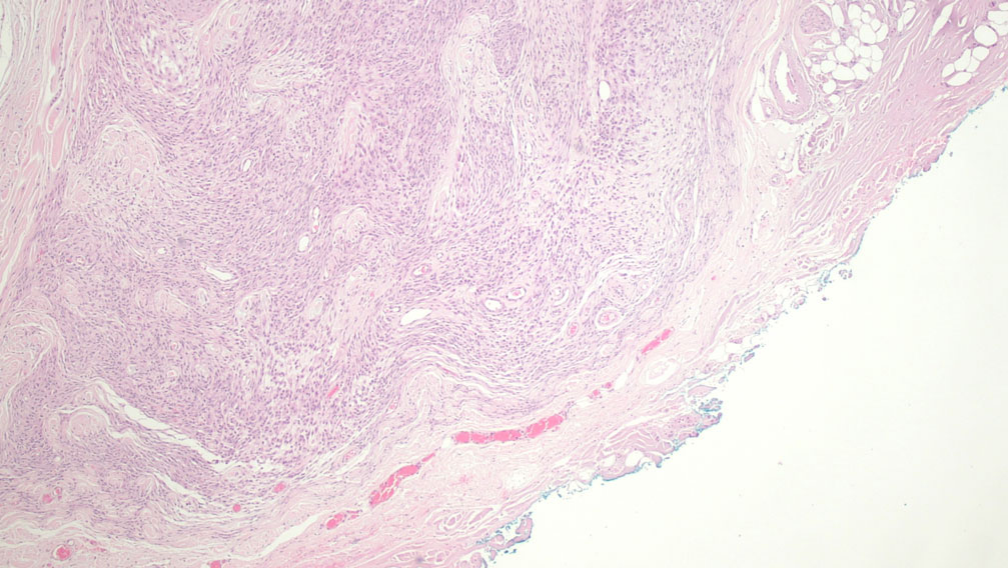
Intermediate grade STS excised with 0‐5 mm lateral margins. Hematoxylin and Eosin stained section showing the deep inked tissue margin composed of dense collagenous fascia (40× magnification). Neoplastic cells were visible within <1 mm of the inked margin without evidence of margin infiltration. With the residual tumor classification scheme, the margin is defined as R0.

Lateral measured surgical margins of 0‐5 mm were reported in 26/47 (55%) surgeries (9/23, 39% MCT, 17/24 STSs, 71%), whereas LMSMs of 6‐10 mm were reported in 21/47 (45%) surgeries (14/23 MCTs, 61%, 7/24 STSs, 29%). In total, 16/47 (34%) excisions returned R1 histological margins, 6/23 (26%) of MCTs, and 10/24 (42%) of STSs (Figure 1). Of masses with R1 margins, 11/16 (69%) had R1 deep histological margins, 12/16 (75%) had R1 lateral histological margins, with 7/16 (44%) of histological margins R1 in both lateral and deep planes. The percentage of R1 histological margins was similar between MCTs and STSs groups for both the deep (5/6 MCTs, 83%, 6/10 STSs, 60%) and lateral histological margins (4/6 MCTs, 67%, 8/10 STSs, 80%). For MCTs, LMSMs of 0‐5 mm resulted in 5/9 (55%) R1 margins, whereas 6‐10 mm LMSMs resulted in 1/14 (7%) R1 margins. For STSs, LMSMs of 0‐5 mm resulted in 7/17 (41%) R1 margins, whereas 6‐10 mm LMSMs resulted in 3/7 (43%) R1 margins.

Histological grades of MCTs and STSs are displayed in Tables 2 and 3. For the MCTs, 12/23 were dermal and 11/23 were subcutaneous. There were no Kiupel high‐grade MCTs, and only 1 high‐grade STS. There was no clear association between the likelihood of R1 margins and histological grade for STSs (8/15 (53%) R1 margins for low grade, 2/8 (25%) for intermediate grade, 0/1 (0%) for high grade). There was also no clear association between the likelihood of R1 margins and histological grade for MCTs (0/2, 0% R1 for Patnaik grade 1, 3/10, 30% for Patnaik grade 2, 3/11, 27% for subcutaneous MCTs).

**TABLE 2:** Mast cell tumor grades

Patnaik grade 1	Patnaik grade 2	Patnaik grade 3	Subcutaneous
10	2	0	11

*Notes*: Grades of tumors excised within the study. Cutaneous tumors as defined by Patnaik.^26^ All cutaneous tumors were low grade using the grading system suggested by Kiupel.^27^

**TABLE 3:** Grades of soft tissue sarcomas excised within the study

Low	Intermediate	High
15	8	1

Binary logistic regression revealed no influence of sex (*P* = .179), age (*P* = .818), bodyweight (*P* = .661), body condition score (*P* = .445), or tumor size (*P* = .253) on the probability of R1 margins. There was also no influence of tumor type (*P* = .484), but an influence of surgical margin (0‐5 mm/6‐10 mm) (*P* = .009) and a tumor type/surgical margin interaction (*P* = .049) on the probability of R1 margins, demonstrating the benefits of a 6‐10 mm lateral surgical margin for MCTs but not STSs. While these data achieved statistical significance, post hoc power analysis demonstrated insufficient statistical power of 0.74 (due to the relatively small group sizes).

**FIGURE 2:**
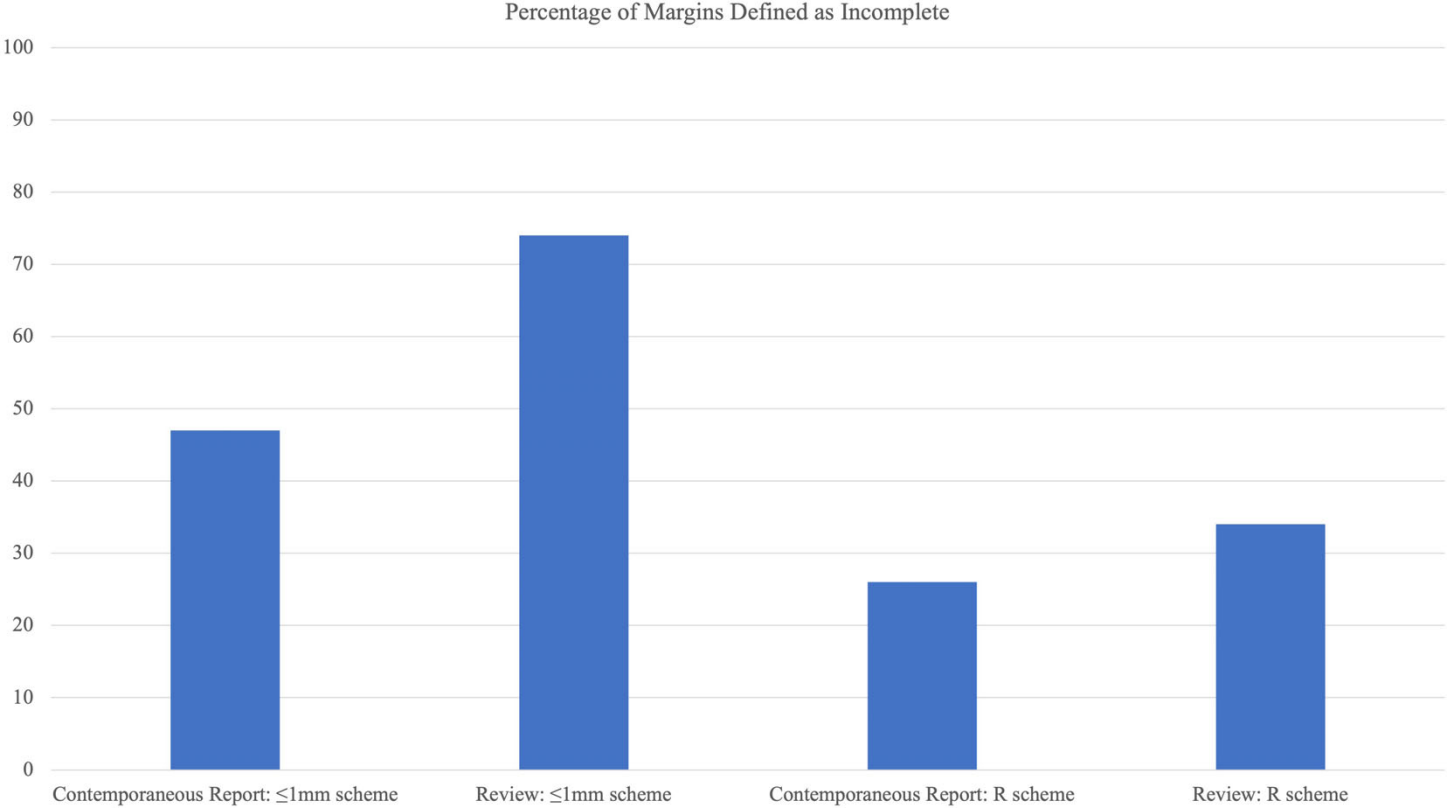
Percentage of excisions classified as incomplete on contemporaneous reports, and after review, with first the ≤1 mm scheme, then the R scheme. The ≤1 mm scheme defines an incomplete margin as tumor cells within ≤1 mm of the inked margin. The R scheme classifies margins as R1 (incomplete) if tumor cells are visible on the inked surgical margin, or R0 (histological tumor‐free margin >0 mm).

Original and reviewed histopathological reports were compared to assess percentage interobserver agreement of the 2 histological reporting schemes. Fewer histological margins were reported as incomplete when the R scheme was used; however, there was greater percentage agreement in histological reporting. When incomplete histological margins were defined with the ≤1 mm scheme, original histological reports reported 47% (22/47) margins as incomplete, while the value was 35/47 (74%) following a retrospective review of the same slides by a single pathologist (percentage agreement 68%). When the R scheme was used, 12/47 (26%) margins were designated R1 on original reports, and 16/47 (34%) R1 following a single pathologist review of the same slides (percentage agreement 83%) (see Figure 2).

Postoperative adverse events occurred in 12/47 (26%) surgeries (4/23, 17% MCTs, 8/24, 33% STSs) and included seroma, superficial surgical site infection, and partial dehiscence; these can be subdivided into 3 grade I outcomes (seroma), 8 grade II outcomes (superficial surgical site infection, or minor dehiscence), and 1 grade III outcome (moderate dehiscence requiring hospitalization for management).

Follow‐up data were available for 36/44 (81%) dogs. The mean and median duration of follow‐up was 844 (± 245) and 807 (range 411‐1254) days respectively. There were 7/36 (19%) dogs deceased at the time of follow up, 3 of unknown causes. Two of the remaining 4 deaths were due to or attributed to the progression of the neoplastic disease. One dog was euthanized due to aspiration pneumonia; the other was euthanized following the death of the owner. Local recurrence (LR) or metastatic disease (MD) was documented or suspected (ie, diagnosis presumed without cytological/histopathological confirmation) in 5/36 (14%) patients (3 local recurrences, 2 regional metastases), 3 of which were STSs (2 LR, 1 MD), 2 MCTs (1 LR, 1 MD). Mean and median times to recurrence were 534 (± 371) and 525 (range 110‐949) days respectively. Of cases with LR/MD, 3/5 (60%) had R1 margins.

Following surgery, 5/47 (11%) of surgical sites subsequently underwent hypofractionated radiotherapy (1/23, 4% MCTs, 4/24, 16% STSs). Of these, 2/5 (40%) had R1 margins and 3/5 (60%) had R0 margins (1 high‐grade STS, 2 intermediate‐grade STSs). Follow‐up data were available for 4/5 (80%) cases undergoing radiotherapy, of which 0/4 (0%) had recurrence within the follow‐up period (mean and median follow up of cases following radiotherapy were 629 (± 142) and 647 (range 412‐811) days respectively). Adjunctive therapy was considered clinically indicated in 12/26 (46%) surgeries with LMSMs 0‐5 mm and clinically indicated in 5/21 (24%) surgeries with LMSMs 6‐10 mm.

## 4 DISCUSSION

Planned narrow excision of peripheral STSs/MCTs, comprising a ≤10 mm margin outside the GVT and a deep fascial plane, resulted in 10/24 (42%) R1 histological margins for PNE of STSs, and 6/23 (26%) R1 histological margins for PNE of MCTs. For MCTs, LMSMs of 0‐5 mm resulted in 5/9 (55%) R1 margins, whereas 6‐10 mm LMSMs resulted in 1/14 (7%) R1 margins. For STSs, LMSMs of 0‐5 mm resulted in 7/17 (41%) R1 margins, whereas 6‐10 mm LMSMs resulted in 3/7 (43%) R1 margins. Surgery was well tolerated, with a moderate incidence of minor complications that did not require additional surgery and a low rate of disease recurrence.

The R scheme was used in this study as an objective method of histopathological margin assessment. The common use of variably wider histological margins to define incomplete excision (eg, ≤1 mm,^7^, ^17^, ^30^ 2 mm,^23^ 3 mm,^12^ 5 mm^31^) conflates histologically tumor‐free excision (absence of microscopic residual tumor at the tissue margins) with histologic safety margin (the minimum histological tumor‐free margin required to significantly reduce the risk of tumor recurrence), but also confuses comparisons between studies, as different outcome measures are being assessed. Histologic safety margins have not been defined in veterinary oncology and are infrequently described in human oncology.^24^ There is also inherent imprecision within a quantified surgical margin, as the measured margin reduces at each stage of processing following surgery (in vivo, ex vivo, postfixation, subgross, when mounted on glass) before a final assessment of the histologically tumor‐free margin,^32^ with a mean length reduction ranging from 35% to 42%.^33^ The greater interobserver percentage agreement in histological reporting when the R scheme was used compared to the ≤1 mm scheme (83% vs 68%), demonstrates the benefit of an objective method of histological margin reporting and merits further investigation in prospective studies.

The intended benefit of a PNE over ME is that it stipulates appropriate oncological technique and an attempt to achieve an R0 deep histological margin, hopefully increasing the likelihood of complete histological excision. Despite surgeons attempting to excise a deep fascial plane below the mass in all surgeries, 23% (11/47) returned R1 deep margins – a similar frequency to the lateral margin, with the frequency similar between STSs and MCTs. This would suggest that an R0 deep fascial plane is not easily achievable when performing PNE of appendicular STSs and MCTs. This may reflect tumor biology or the inherent limitations of achieving a deep fascial plane in appendicular sites. Soft tissue sarcomas tend to grow asymmetrically beyond the GVT along fascial planes, meaning that the GVT does not often represent the histological limits of neoplasia.^23^ Therefore, the frequency of R1 histologic margins following PNE of STSs will depend largely on tumor biology rather than the width of surgical excision.^21^

Peripheral tumor location (particularly the hind limb) has been associated with an increased likelihood of incomplete histological margins and recurrence in both STSs and MCTs.^5^, ^34^ It has also been shown that fascial integrity is lacking in the distal antebrachium. An appendicular deep fascial plane may therefore be either a poor barrier to the spreading of a tumor or challenging to surgically achieve.^35^ This combination of factors perhaps explains the similar frequency of R1 deep margins across tumor types, suggesting that even more extensive resections may struggle to result in complete histological excision in appendicular oncological surgeries. Despite this, the success of PNE in achieving R0 deep margins in 36/47 (77%) of surgeries supports our prior observation that there are benefits to adhering to oncological principles when performing appendicular oncological resections, even if compromises to the lateral margins have been necessary.

Planned narrow excision of MCTs was described for the first time in this study. A recent systematic review assessing margins for removal of cutaneous MCTs in dogs^36^ identified 4 relevant articles, of which only 1 described the use of a margin of <2 cm (proportional margins).^7^ This is a technique not always feasible for appendicular MCTs, due to the limited availability of skin for closure. Studies reporting histological margins following MCT excision have used wide surgical margins (9% incomplete),^30^ proportional surgical margins (Pratschke^7^ and Itoh^6^ found 14% and 8% complete respectively), or retrospectively assessed a 10 mm surgical margin from a mass excised with wider (eg, 20/30 mm) margins (22% incomplete).^5^ As an incomplete margin was arbitrarily defined as 1 mm in these papers, results cannot be directly compared with our data. However, a recent paper assessing the frequency of incomplete histological margins when performing ME of MCTs reported 88% of histological margins as incomplete (defined as tumor on ink, so analogous to R1 margins). This was worse than our overall frequency of 16/47 (34%) R1 margins, thereby demonstrating a clear benefit of PNE over ME in MCTs.^29^ Furthermore, when 6‐10 mm LMSMs were used for MCTs, we found an R1 margin frequency of 1/14 (7%), whereas when LMSMs of 0‐5 mm were used, 5/9 (55%) margins were R1, with a benefit for performing PNE for MCTs with this wider LMSM (*P* = .049). Further studies, ideally prospective, are therefore needed to assess whether the distinction between 0‐5 and 6‐10 mm LMSMs represents an important minimum excision margin for achieving R0 histologic margins in appendicular low‐grade and subcutaneous MCTs.

A proportion of the MCTs described in this paper were subcutaneous. Subcutaneous MCTs are considered by some authors to behave similarly to Patnaik grade II^26^ cutaneous MCTs and are therefore often grouped with cutaneous MCTs for analysis.^37^ However, it is accepted that subcutaneous mast cell tumors are not completely analogous to their cutaneous counterparts, as they are considered to respond more favorably to surgery than grade II MCTs, with lower frequencies of local recurrence.^38^ While this may have affected the assessment of clinical outcomes compared to other studies, it was considered unlikely that the inclusion of subcutaneous MCTs would bias the primary study objective. Subcutaneous and cutaneous MCTs are approached similarly by the oncological surgeon, with the distinction often being made histologically rather than before surgery. It was therefore considered appropriate to include them.^37^, ^39^, ^40^

Unlike MCTs, there was no association found between the percentage of R1 histological margins for 0‐5 mm/6‐10 mm LMSMs for PNE of STSs. Reported percentages of incomplete margins following marginal excision of peripheral STSs range from 36% to 100%,^11^, ^12^, ^17^ while percentages following wide excision are reported as 0%‐54%.^10^, ^22^ As these studies include variable definitions of what constitutes an incomplete margin (not defined,^10^, ^11^, ^22^ tumor on ink,^12^ tumor <1 mm from ink^17^), exact comparisons are not possible. However, the frequency of R1 margins (10/24, 42%) was broadly consistent with the reported range. The frequency of recurrence of STSs was 3/18 (17%), which is not dissimilar to the frequency of 23% described by McSporran et al (majority unplanned excisions managed in general practice with a whole‐body distribution),^17^ or 21% reported by Bray et al. (majority unplanned excisions managed in general practice with a whole‐body distribution),^41^ despite containing a tumor population at greater risk of recurrence compared to some other sites.^34^ However, the proportion of high‐grade tumors was lower in our study (4%) than these others (6%,^41^ and 7%^17^) and would therefore be expected to reduce the risk of recurrence in our population of cases. Wider resection should be the treatment of choice for the management of STSs if possible; however, the acceptable frequency of complications following primary wound closure, and low rates of recurrence, means that for appropriate cases, PNE has advantages over other techniques (eg, radical resection,^22^ wide excision with second intention healing^10^).

A key justification for PNE is that it achieves limb salvage while maintaining the option for adjunctive therapy if indicated. The low frequency of subsequent adjuvant therapy reported in this study (5/47, 11%) reflects the “real‐world” clinical picture, which incorporates multiple factors such as cost, prognosis, willingness to tolerate adverse effects, and convenience of treatment. It is therefore more useful to consider whether adjunctive therapy was considered clinically indicated. Adjunctive therapy was clinically indicated in 17/47 (36%) of all PNE: in 12/26 (46%) of cases when LMSMs of 0‐5 mm were attempted, 5/21 (24%) of cases when LMSMs of 6‐10 mm were attempted. As these figures ignore other clinical factors (eg, low tumor grade, mitotic index, and tissue composition of the deep margin), which may further reduce the justification for adjunctive therapy, these values can be taken as maximums, and may therefore influence decision making for clients and clinicians when considering the merits of PNE.

This study was based upon retrospective data, and therefore suffered from many of the limitations of this study design. Data were reliant on clinical notes, and therefore recorded variables such as margin and tumor size were based on surgical reports and clinical notes without standardization of measurements etc. Advanced imaging was not routinely performed, which may have led to an underestimation of the gross tumor extent and potential unintentional intralesional excisions. Follow‐up data were based upon referring veterinarian reports and there was no further investigation (eg, cytology or staging) of suspected recurrences, leading to potential sources of bias. The 12‐month minimum follow‐up obtained was a compromise to achieve higher case numbers; however, it will not have been sufficient to identify all cases of recurrence in this population, as reported median times to recurrence for MCTs and STSs are greater than this.^22^, ^42^ With low numbers of high‐grade tumors (1/24 STSs, 0/23 MCTs), this study had insufficient data to demonstrate differences in outcome between different histological grades. The low numbers of recurrence also mean that the association of R1 margins with recurrence could not be evaluated, and further studies are warranted to investigate this. Pooling groups of MCTs (subcutaneous and cutaneous) and STSs together for analysis reflect a similar clinical presentation and decision‐making process for clinicians and was considered an appropriate approach. Finally, while case numbers were sufficient for comparison between MCTs and STSs and historic controls, numbers were smaller for comparison of surgical margins within these groups. Further studies with greater numbers are needed to confirm these findings (as demonstrated by the post hoc analysis). Narrower margins were also more commonly performed, due to the nature of these tumors, leading to potential type II errors.

In conclusion, PNE comprising a ≤10 mm lateral margin and a deep fascial plane is a useful technique for histopathological diagnosis and management of peripheral STSs and MCTs when other options are not available. For MCTs, the technique appears to be more effective when a 6‐10 mm lateral margin is used. The use of the R scheme resulted in increased interobserver percentage agreement in histological margin reporting and warrants further research. Prospective studies are needed to understand clinical outcomes with the PNE technique better for appendicular MCTs and STSs; however, in this cohort of cases, PNE appears to be a reasonable surgical approach for limb salvage.

## CONFLICT OF INTEREST

The authors declare no conflicts of interest related to this report.
